# Randomized multicenter noninferiority phase III clinical trial of the first biosimilar of eculizumab

**DOI:** 10.1007/s00277-021-04624-7

**Published:** 2021-08-16

**Authors:** Alexander D. Kulagin, Vadim V. Ptushkin, Elena A. Lukina, Igor L. Davydkin, Alexander V. Korobkin, Vladimir S. Shamrai, Tatyana S. Konstantinova, Tatyana S. Kaporskaya, Tatyana A. Mitina, Tatyana I. Ksenzova, Evgeny V. Zuev, Oksana A. Markova, Elena V. Gapchenko, Dmitry A. Kudlay

**Affiliations:** 1 RM Gorbacheva Research Institute, Pavlov University, 6/8 L’va Tolstogo St, Saint Petersburg, 197022 Russia; 2 Botkin Moscow City Clinical Hospital, Moscow, Russia; 3 National Medical Research Center for Hematology, Moscow, Russia; 4 Samara State Medical University, Samara, Russia; 5 Chelyabinsk Regional Clinical Hospital, Chelyabinsk, Russia; 6 Rostov Regional Clinical Hospital, Rostov-on-Don, Russia; 7 Sverdlovsk Regional Clinical Hospital No. 1, Yekaterinburg, Russia; 8 Irkutsk Regional Clinical Hospital, Irkutsk, Russia; 9 Moscow Regional Clinical Research Institute Named After M.F. Vladimirsky, Moscow, Russia; 10 Tyumen Regional Clinical Hospital No. 1, Tyumen, Russia; 11 JSC GENERIUM, Volginsky Settlement, Vladimir Region, Russia; 12 I.M. Sechenov First Moscow State Medical University (Sechenov University), Moscow, Russia

**Keywords:** Paroxysmal nocturnal hemoglobinuria, Eculizumab, Biosimilar, Efficacy, Safety

## Abstract

Currently, eculizumab is the main effective treatment for paroxysmal nocturnal hemoglobinuria (PNH). The aim of this randomized multicenter noninferiority study was to evaluate the efficacy and safety of the Biosimilar (Elizaria) in comparison with the Originator (Soliris) in patients with PNH. Biosimilar and Originator were administered at a dose of 600 mg weekly for 4 weeks at the initial stage in naive patients, as well as for maintenance therapy at a dose of 900 mg every 2 weeks in all patients. The primary endpoint was a comparative assessment of hemolytic activity based on the area under the lactate dehydrogenase (LDH) concentration–time curve during the maintenance therapy. Thirty-two (32) patients were randomized for therapy with Biosimilar (*n* = 16) or Originator (*n* = 16). The mean values of LDH concentration–time curve were similar in both treatment groups without statistically significant differences (*p* > 0.05). Evaluation of secondary endpoints has shown no statistically significant differences between the groups. Safety values were comparable in both treatment groups. The data obtained confirm that the Biosimilar is not inferior to the Originator in terms of the main efficacy parameter, and is also comparable with it in terms of safety and additional efficacy parameters. Clinicaltrials.gov identifier: NCT04463056

**Supplementary Information:**

The online version contains supplementary material available at 10.1007/s00277-021-04624-7.

## Introduction

Paroxysmal nocturnal hemoglobinuria (PNH) is a rare clonal hematological disease that develops as a result of the clonal expansion in one or more clones of hematopoietic stem cells due to the somatic mutation of the *PIG-A* gene localized on the active X chromosome [1]. This genetic defect results in the disturbance of early synthesis of the carbohydrate branch of glycosylphosphatidylinositol (GPI) anchor involved in the fixation of a number of molecules that protect blood cell membranes against the damaging effect of the complement system (CD55, CD59). Activation of the complement system leads to the C5b protein formation, which is a key subunit in the formation of a membrane attack complex (MAC) followed by blocking of the complement inhibitor CD59 [2–4]. MAC is a key factor causing intravascular hemolysis, platelet activation, and formation of procoagulant membrane microparticles [5–9].

The current pathogenetic treatment for PNH-associated intravascular hemolysis is eculizumab, a humanized monoclonal antibody against complement component C5. In most patients, eculizumab treatment reverses all intravascular hemolysis manifestations, prevents severe complications, and, eventually, normalizes survival and quality of life of PNH patients [10, 11].

Elizaria (eculizumab), developed by the Russian biotechnological company JSC GENERIUM, was registered in the Russian Federation in 2019 for pathogenetic therapy of patients with PNH and atypical hemolytic uremic syndrome as a Biosimilar to the original product Soliris (Alexion Pharma GmbH).

At all stages of the development and production of the Biosimilar, a comprehensive study of the quality, safety, and efficacy of the medicinal product was performed in accordance with the regulatory requirements of the Eurasian Union (EAEU) and the European Medicines Agency (EMA) [12–14]. During the development of the Biosimilar, in vitro and in vivo preclinical studies were performed in comparison with the Originator (Soliris, Alexion Pharma GmbH, Switzerland). The results have shown the equivalence of medicinal products in terms of in vitro activity and toxicity. The phase I clinical study involving 30 healthy volunteers provided comparable safety and tolerability data for the Biosimilar and Originator. In the phase Ib study in 11 PNH patients, the pharmacokinetic and pharmacodynamic properties of the Biosimilar were studied, confirming its efficacy and demonstrating the safety [15].

The phase III ECU-PNH-III study (clinicaltrials.gov identifier: NCT04463056) was conducted to show that the investigational medicinal product is not inferior to the Originator in terms of area under the LDH concentration–time curve (LDH AUC) (noninferiority design). The results of the study were partially reported at the 2019 ASH Annual Meeting [16]. All clinical trial activities were funded by JSC GENERIUM.

## Methods

### Patients

The study was conducted at 10 accredited medical centers in Russia and included 32 patients from 23 to 71 years of age, with PNH confirmed by flow cytometry to assess the size of the PNH clone among red blood cells (RBC), granulocytes, and monocytes, with intravascular hemolysis and concomitant clinical symptoms at present or in past medical history with or without eculizumab administration. PNH clone testing was performed according to the previously described protocol of high-sensitivity flow cytometry [17]. All patients were vaccinated against meningococcal infection with a tetravalent vaccine (Neisseria meningitidis serotypes A, C, Y, and W135). For naive patients, it was obligatory to exceed the LDH upper limit of normal by 1.5 times. The key exclusion criteria were the presence of diseases associated with bone marrow failure with the PNH clone (aplastic anemia, myelodysplastic syndrome, idiopathic myelofibrosis), previous infectious diseases caused by Neisseria meningitidis, active nonspecific infectious diseases, and completion of eculizumab treatment less than 70 days before the enrollment. Informed consent was obtained from all patients for being included in the study prior to any procedure.

### Study design

A randomized, open-label, comparative, multicenter study was approved by the Ministry of Health of Russia (No. 546 dated October 17, 2017) and the Ethics Committee of MoH (No. 153 eff. date August 22, 2017) and was conducted in accordance with the ethical standards of the responsible committees on human experimentation (national and local) and with the Helsinki Declaration of 1975, as revised in 2013. The study consisted of the following periods: Screening up to 4 weeks, Treatment (26 weeks), and Follow-up (2 weeks).

### Treatment

Patients were allocated by variable block size (4 and 6 block random allocation sequence was generated by biostatistician in Stata14) randomization using the IVRS system with a 1:1 ratio into two groups: Group A, in which patients received the Biosimilar (Elizaria), and Group B, in which patients received the Originator (Soliris). Preliminary stratification was performed depending on the status of previous treatment with eculizumab (patients not receiving eculizumab/patients receiving a maintenance dose of eculizumab before enrollment in the study). Eculizumab-naive patients in both groups received the initial cycle of therapy consisting of four weekly intravenous administrations of the medicinal product at a dose of 600 mg, with subsequent maintenance therapy at a dose of 900 mg every 2 weeks. Patients previously treated with eculizumab started the treatment at the maintenance dose.

### Investigations

Evaluation of the pharmacokinetic (PK) parameters of the medicinal product was performed based on the determination of total (free and bound) eculizumab by biolayer interferometry (Octet® QKe System (Pall ForteBio)) with using Octet® Software, v.10.0 (Pall ForteBio). The following basic pharmacokinetic (PK) parameters were calculated: minimum product concentration (C_min_), maximum product concentration (C_max_), minimum product concentration at the end of the dosing interval after establishing a stationary distribution (C_trough_), area under the concentration–time curve throughout the dosing interval after establishing a stationary distribution (AUC_t,ss_). As additional PK parameters, the time to reach the maximum product concentration (T_max_), elimination constant (K_el_), medicinal product elimination half-life (T_1/2_), mean retention time of medicinal product in the systemic circulation (MRT), clearance (Cl), and stationary volume of distribution (V_ss_) were calculated.

The analysis of pharmacodynamic (PD) parameters included evaluation of the membrane attack complex concentration in the blood serum (MAC, C5b-9) by the enzyme-linked immunosorbent assay (MicroVue Complement SC5b-9 Plus EIA MicroVue Complement SC5b-9 Plus EIA (Quidel, USA)) at the same time periods as for the PK assessment.

The safety of therapy was assessed by the incidence and severity of adverse drug reactions (ADRs) according to symptoms, physical examination, assessment of vital signs, electrocardiography (ECG), laboratory and instrumental studies, and patient diaries.

Immunogenicity was determined by the level of anti-drug antibodies, including the neutralizing activity of antibodies to eculizumab, by the method based on the bridging enzyme-linked immunosorbent assay. The evaluation was performed at 1, 5, 13, 21 weeks of treatment and after the end of the treatment.

All data was collected using the platform EDC system CSOnline version 7.5.501.1 by Ennov clinical®.

### Endpoints

Comparative evaluation of hemolysis activity based on the area under the LDH concentration–time curve (LDH AUC) during the maintenance therapy with the investigational or reference product was the primary endpoint of the study. The secondary points included the area under the LDH concentration–time curve (LDH AUC) during 26 weeks of treatment, the number of packed red blood cells (pRBC) transfusions performed, the change in the values of the Functional Assessment of Chronic Illness Therapy-Fatigue Scale (FACIT-Fatigue) and the European Organization for Research and Treatment of Cancer Quality of Life Questionnaire (EORTC QLQ-C30), the number/proportion of patients with various thrombotic complications, patients requiring pRBC transfusions, and patients with breakthrough hemolysis.

### Statistical analysis

The standard methods available in Stata (StataCorp LLC), version 14, were used to analyze the data. The PK and PD data analysis was performed using the PkSolver software. LDH AUC during the period of maintenance therapy with the investigational or reference product was performed to assess the primary efficacy endpoint in terms of chronic hemolysis in a PP population. To demonstrate the difference in LDH AUC between the groups, a 95% confidence interval was built, which should not have crossed the noninferiority boundary, which is set in this study as 50% of the previously identified placebo-controlled effect at a value of 150,635 U/L*days[18]. The *χ*2 criterion or Fisher’s exact test was used to analyze the secondary efficacy parameters, which are categorical variables. Descriptive statistics was used to analyze the secondary efficacy parameters, which are quantitative variables. For the intergroup analysis, Student’s *t*-test or Mann–Whitney test was used, depending on the type of data distribution. A normality test was performed using the Shapiro–Wilk test.

## Results

### Patients

**Fig. 1:**
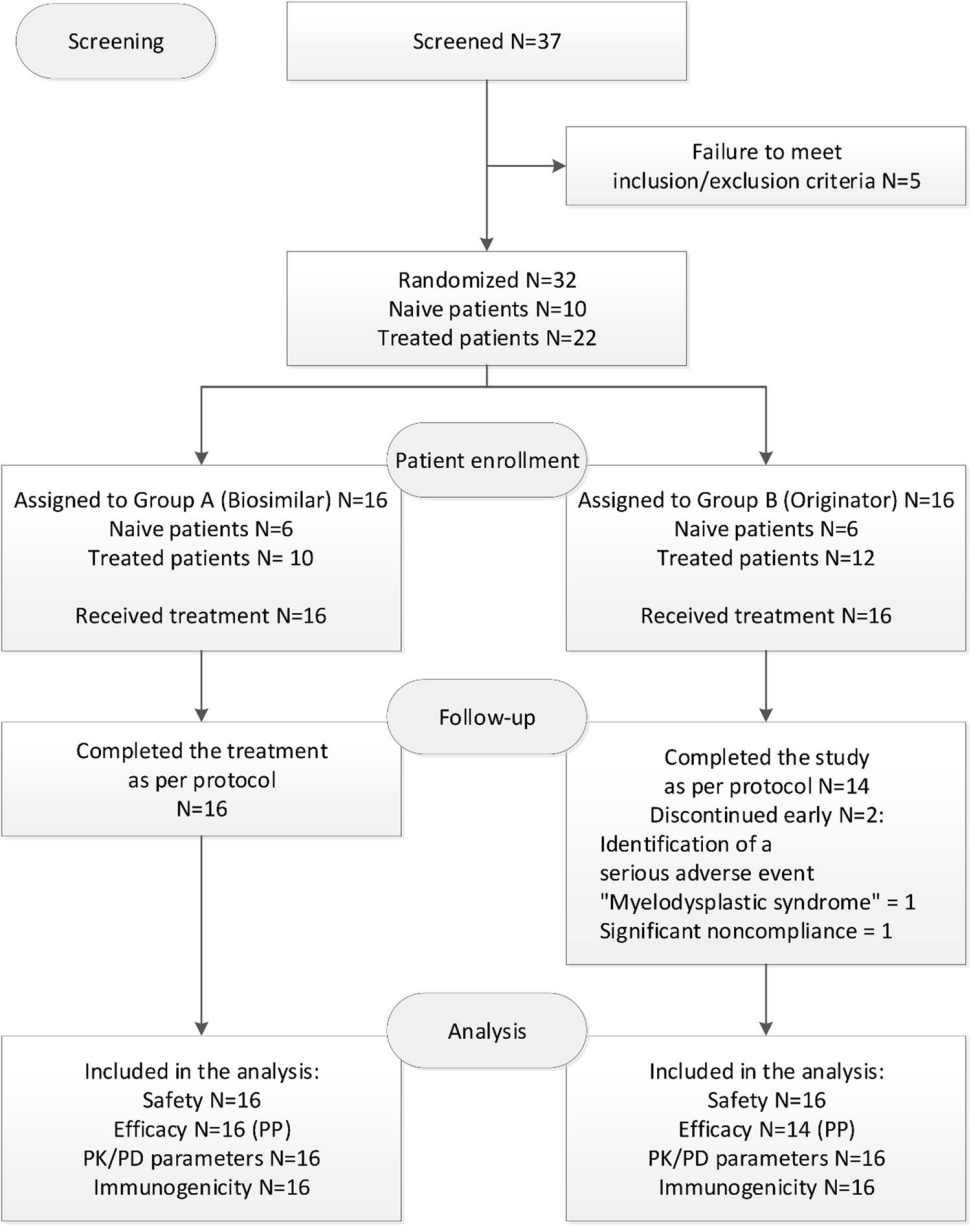
Distribution of patients in the study

Investigators screened 37 patients, 32 of whom (86.5%) met all the inclusion/exclusion criteria and were randomized into two treatment groups. Twenty-two of the 32 randomized patients (68.9%) had previously received treatment with Soliris, and 10 patients (31.3%) had not received prior treatment with eculizumab. Two patients discontinued the study early and were excluded from the PP population due to the development of a serious adverse event “Myelodysplastic syndrome,” and because of significant violations of the study protocol (Fig. 1). The median age of patients was 39 years in Group A and 36.5 years in Group B (Table 1). The study included 15 male patients and 17 female patients. For most of the baseline characteristics, the patients of both groups were comparable. There was a significantly higher LDH level in previously untreated patients and probably due to this reason a lower GFR in Group A.

**Table 1:** Baseline characteristics of the patients enrolled

*Variable*	Group A (*Biosimilar*) (*N* = 16)	Group B (*Originator*) (*N* = 16)	*P*-value
***Age – years***			0.771
*Median*	39	36.5	
*Q1–Q3*	30.5–58	30–58	
***Female sex – no. (%)***	7 (43.8)	10 (62.5)	0.288
***White race – no.***^***†***^	16	16	1.000
***History of thrombosis – no. (%)***	2 (12.5)	4 (25)	0.327
***History of aplastic anemia – no. (%)***	5 (31.3)	1 (6.3)	0.086
***Lactate Dehydrogenase***			
*Naive Median – U/liter*	2,121	538	0.057
*Q1–Q3*	1,657–2,811	397.5–1,348	
*Treated Median – U/liter*	330	224	0.072
*Q1–Q3*	297–401	204–282	
***Red blood cells PNH clone (II* + *III type) – %***			0.223
*Median*	68.7	55.5	
*Q1–Q3*	36.8–92.2	24.3–65.4	
***Granulocyte PNH clone – %***			0.462
*Median*	94.85	95.55	
*Q1–Q3*	85.7–97.9	71.0–97.3	
***Hemoglobin – g/liter***			0.353
*Median*	93.5	100.5	
*Q1–Q3*	78.5–115	90–126	
***Glomerular* f*iltration* r*ate, mL/min/1.73***^***2***^			0.0253
*Median*	86.8	117.1	
*Q1–Q3*	67.1–111.7	94.5–122.1	
***Mean pulmonary pressure – mmHg***			0.808
*Median*	22.5	22.5	
*Q1–Q3*	10–26.5	10–25	
***EORTC QLQ-C30 – points***			0.375
*Median*	49.4	54.4	
*Q1–Q3*	48.3–53.9	48.9–57.2	
***FACIT-Fatigue – points***			0.067
*Median*	36	40.5	
*Q1–Q3*	27–40.5	32.5–47.5	
***MAC – ng/mL***			0.109
*Median*	237.05	161.87	
*Q1–Q3*	171.13–843.67	133.81–217.34	

^†^Race information provided by patients themselves

Notes: *FACIT-Fatigue*, Functional Assessment of Chronic Illness Therapy – fatigue scale; *EORTC QLQ-C30*, European Organization for Research and Treatment of Cancer Quality of Life Questionnaire; *MAC*, membrane attack complex

### Pharmacokinetics

**Fig. 2:**
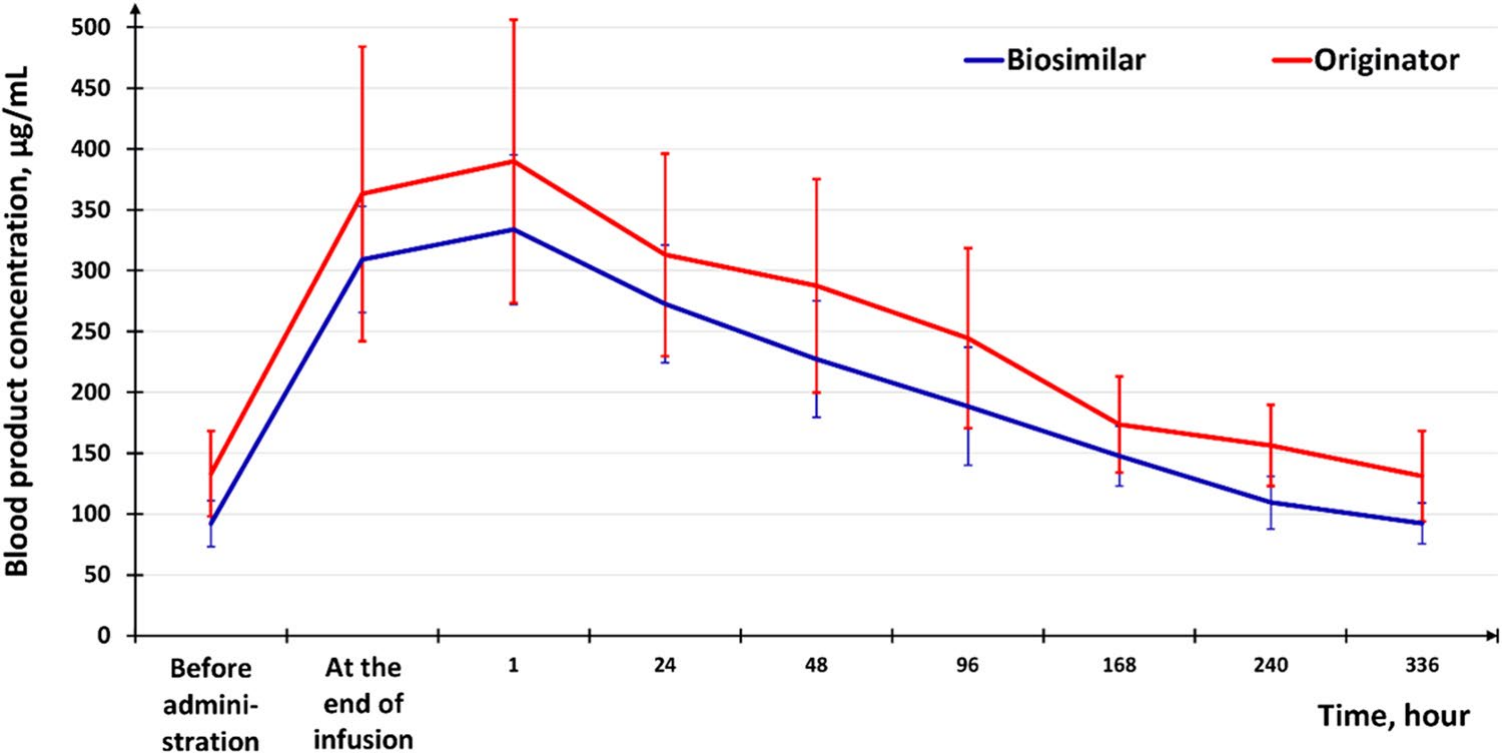
Mean pharmacokinetic curves for the recorded eculizumab serum steady-state concentration in the extensive examination

Mean eculizumab steady-state concentration 5 min before the administration of the Biosimilar or Originator was 92.16 ± 38.59 μg/mL in Group A and 133.21 ± 71.56 in Group B. There were no statistically significant differences between the groups in eculizumab concentration (*p* > 0.05) at the majority of the time points. Both PK curves are in good agreement: the concentration of the medicinal product increases significantly after the end of the infusion, reaches a maximum at “1 h after the end of infusion” point, and then gradually decreases (Fig. 2). Intergroup comparison of the PK parameters revealed no statistically significant differences (*p* > 0.05) (Table 2).

**Table 2:** Characteristics of pharmacokinetic parameters in both groups

PK parameter	Parameter	Group A (Biosimilar)	Group B (Originator)	*P* (mu)
C_min_, µg/mL	M	64.89	94.13	0.065
	SD	25.96	58.76	
C_max_, µg/mL	M	351.35	407.45	0.635
	SD	114.44	263.63	
C_trough_, µg/mL	M	92.19	131.16	0.075
	SD	33.26	73.49	
AUC_t,ss_, µg*h/mL	M	53,579.37	70,612.81	0.123
	SD	20,853.74	33,648.98	
T_**max**_, h	M	4.26	1.26	0.415
	SD	7.83	0.55	
K_el_, h^−1^	M	0.0034	0.0028	0.236
	SD	0.0016	0.0011	
T_1/2_, h	M	375.08	277.00	0.236
	SD	598.62	83.75	
MRT, h	M	536.63	408.15	0.206
	SD	858.25	135.57	
CL, mL/h	M	11.97	8.50	0.053
	SD	6.14	3.56	
V_ss_, L	M	3.80	3.29	0.385
	SD	1.23	1.19	

Notes: *C*_*min*_, minimum product concentration; *C*_*max*_, maximum product concentration; *C*_*trough*_, minimum product concentration at the end of the dosing interval after establishing a stationary distribution; *AUC*_*t,ss*_, area under the concentration–time curve during the dosing interval after establishing a stationary distribution; *T*_*max*_, h, time to reach the maximum product concentration; *K*_*el*_, elimination constant; *T*_*1/2*_, half-life; *MRT*, mean retention time; *CL*, clearance; *V*_*ss*_, stationary specific volume of distribution

### Pharmacodynamics

**Fig. 3:**
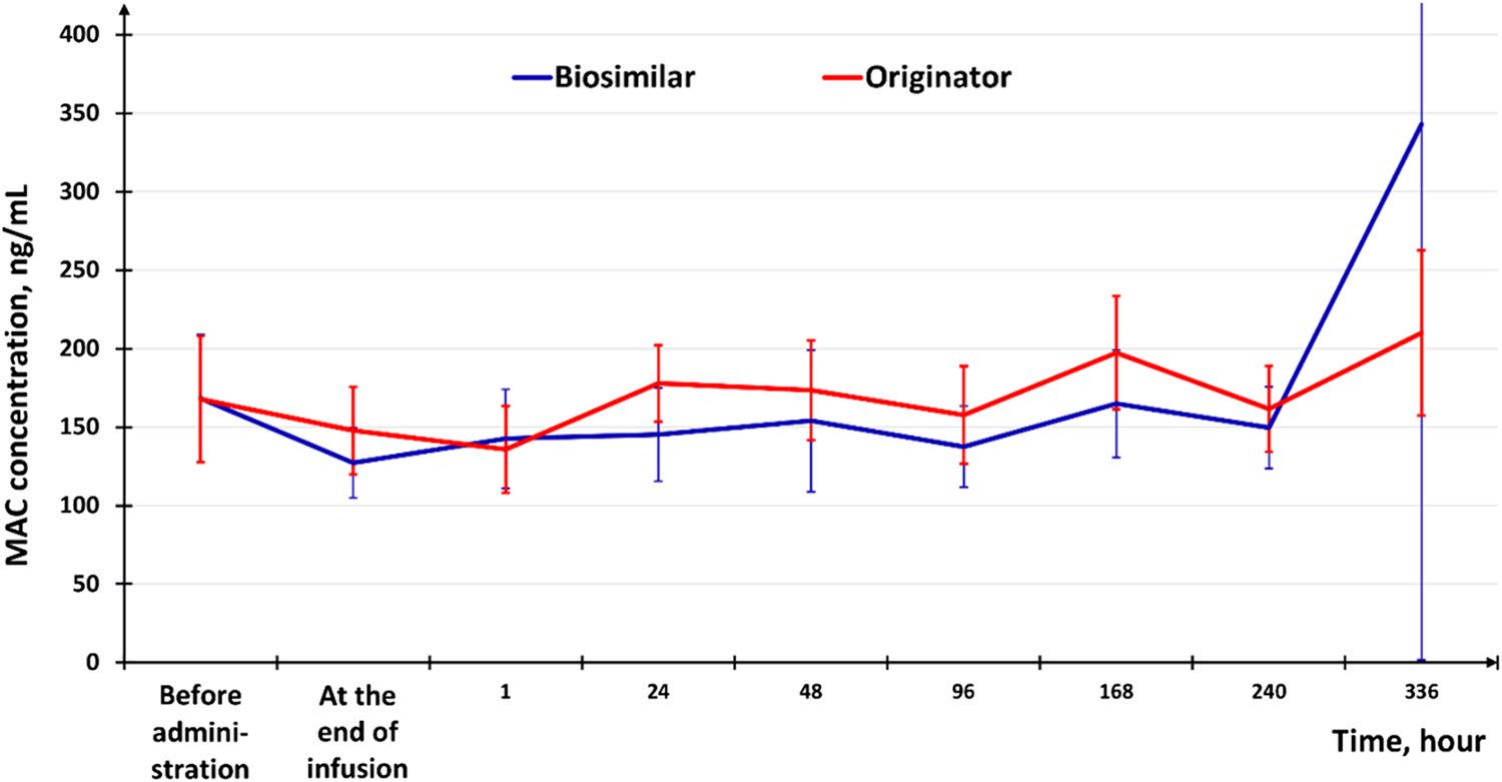
Mean pharmacodynamic curves for the recorded membrane attack complex values (ng/mL) in steady state in the extensive PK study

There were no statistically significant differences between the groups in MAC concentration (*p* > 0.05) at the majority of the time points. One *p*-value ranged from 0.01 to 0.05 (Visit 11, point 1 h after the end of the infusion, *p* = 0.0237), which, given the multiplicity of comparisons, also cannot be considered statistically significant differences. Both PD curves are in good agreement: MAC concentration decreases upon the medicinal product infusion completion, then it gradually increases, and reaches its maximum at the end of the dosing interval (Fig. 3).

### Efficacy

**Fig. 4:**
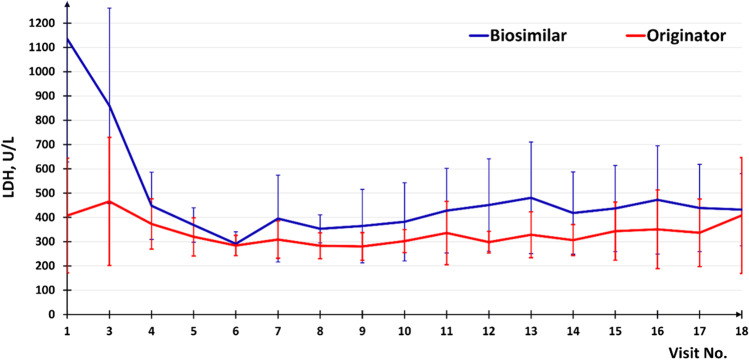
Dynamics of lactate dehydrogenase activity during the study (95% CI for the mean)

Similar LDH AUC values were observed in both treatment groups, given the high coefficient of variation. Average LDH AUC values were 62,957.6 ± 46,066.5 U/L*days (95% CI [38,410.4; 87,504.7]) in Group A and 49,702.6 ± 26,182.1 U/L*days (95% CI [34,585.5; 64,819.7]) in Group B (*p* = 0.351). Point estimation of the intergroup difference in LDH AUC amounted to 13,255.0 U/L*day (one-sided 95% CI [− 10,492.9; 37,002.8]) (Fig. 4).

Estimation of the area under the LDH concentration–time curve (LDH AUC) between 1 and 26 weeks of treatment showed results similar to the primary endpoint, taking into account the high coefficient of variation, the LDH AUC value in the two groups (Table 3).

**Table 3:** The results of a comparative assessment of the secondary efficacy endpoints in the compared groups

Secondary efficacy criterion	Biosimilar	Originator	*P*-value
The area under the curve throughout the study, U/L*day			
M ± SD	75,670.1 ± 49,432.2	59,254.0 ± 30,396.9	0.291
Me; IQR	66,991.25; 24,058.75	52,915.75; 21,702.5	
95% CI	(49,329.5; 102,010.6)	(41,703.3; 76,804.6)	
Changes in hemoglobin level during the maintenance therapy period, g/L			
M ± SD	2.4 ± 14.3	2.8 ± 13.1	0.949
Me; IQR	1; 15	4; 20	
**95% CI**	(− 5.2; 10.1)	(− 4.2; 9.7)	
*Patients with stable hemoglobin level or its increase during the maintenance therapy period*	12/16 (75%)	9/14 (64.3%)	0.585
*Patients with various thrombotic complications*	0/16 (0%)	1/14 (7%)	1.000
*Patients requiring transfusions of RBC-containing donor blood components*	2/16 (12.5%)	3/14 (31.3%)	0.642
*Patients with breakthrough hemolysis*	3/16 (18.8%)	0/14 (0%)	0.228
Change in the PNH RBC clone size at Week 26 of treatment in naive patients, %			
M ± SD	11.0 ± 21.61	11.60 ± 9.19	0.972
Me; IQR	8.7; 33.3	11.6; 13.0	
95% CI	(− 11.68; 33.68)	(− 70.99; 94.19)	
Change in the PNH granulocyte clone size at Week 26 of treatment, %			
M ± SD	− 0.55 ± 4.89	4.17 ± 11.85	0.228
Me; IQR	0.3; 3.7	2.0; 3.5	
95% CI	(− 3.16; 2.05)	(− 2.67; 11.01)	
Changes in FACIT-Fatigue score			
M ± SD	8.1 ± 8.7	2.3 ± 6.6	0.053
Me; IQR	7; 9.5	4; 9.0	
95% CI	(3.4; 12.7)	(− 1.5; 6.1)	
Changes in EORTC QLQ-C30 score			
M ± SD	1.67 ± 3.90	− 0.32 ± 4.47	0.204
Me; IQR	2.2; 5.3	− 0.3; 3.9	
95% CI	(− 0.41; 3.74)	(− 2.90; 2.26)	

Notes: *FACIT-Fatigue*, Functional Assessment of Chronic Illness Therapy – fatigue scale; *EORTC QLQ-C30*, European Organization for Research and Treatment of Cancer Quality of Life Questionnaire; *LDH AUC*, area under the LDH concentration–time curve

Comparable positive mean values of hemoglobin level changes during the period of maintenance therapy were noted in the both groups and amounted to 2.4 ± 14.3 g/L in Group A and 1.6 ± 12.2 g/L in Group B.

**Fig. 5:**
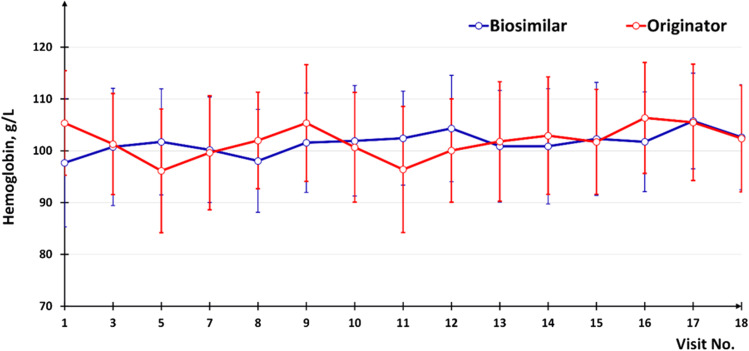
The dynamics of the hemoglobin level during the study period (error bar shows 95% CI for the mean)

Hemoglobin level stabilization (fluctuations not exceeding 5 g/L) during the maintenance therapy period was achieved in 6 of 16 patients (37.5%) who received the Biosimilar and in 2 of 14 patients (14.3%) who received the Originator. In total, stabilization or increase in hemoglobin level was observed in 12 of 16 patients (75.0%) who received the Biosimilar, and in 9 of 14 patients (64.3%) who received the Originator (Fig. 5). Analysis of data in terms of the number and proportion of patients who needed pRBC transfusions, as well as in terms of the number of transfusions, revealed no statistically significant differences between the groups. In Group A, 2 of 16 patients (12.5%) required blood transfusions, while there were 3 of 14 such patients (21.4%) in Group B.

One thrombotic complication (thrombophlebitis of the cubital vein, at the medicinal product infusion site) was recorded in Group B. During the entire study period, 4 cases of breakthrough hemolysis were recorded in 3 of 16 patients (18.8%) in Group A. The change in the mean value of PNH clone of type II + III RBC and granulocytes in naive patients relative to the baseline value was similar in both groups.

The change in the total score of the questionnaires used was statistically insignificant in the compared groups. Thus, the mean value of the change in the total score of the EORTC QLQ-C30 at the end of the study relative to the baseline value at the screening amounted to 1.67 ± 3.90 points in Group A and − 0.32 ± 4.47 points in Group B. Meanwhile, the mean values of the change in the total score of the FACIT-Fatigue at the end of the treatment period relative to the baseline value amounted to 8.1 ± 8.7 points in Groups A and 2.3 ± 6.6 points in Group B.

### Safety

A total of 13 ADRs were reported after the administration of the Biosimilar in 2 patients and Originator in 3 patients. They were associated with investigations, blood and lymphatic system disorders, infectious and parasitic diseases, kidney and urinary tract disorders, general disorders and administration site conditions, and metabolic and nutritional disorders, and had potential, probable, or definitive relation to the investigational medicinal products (Table 1 in supplement).

### Immunogenicity

According to the immunogenicity analysis, no statistically significant differences between the groups in terms of anti-drug antibodies (ADAs) detection frequency were identified at any of the Study Visits (*p* > 0.05). ADAs detected in two patients in Group A (Biosimilar) were formed as a result of previous therapy with the original medicinal product and did not have neutralizing activity. New cases of ADA formation were not revealed during the study.

## Discussion

In this study, the noninferiority assessment of the efficacy of the first Biosimilar (Elizaria) relative to the Originator in treating PNH patients was made to confirm biosimilarity [12, 13]. The result of the primary endpoint analysis (LDH AUC during maintenance therapy) showed that the Biosimilar is not inferior to the Originator in terms of efficacy. Thus, the point estimate of the LDH AUC intergroup difference was 13,255.0 U/L*days (one-sided 95% CI [− 10,492.9; 37,002.8]), that is, the specified confidence interval did not cross the specified limit of noninferiority. In the PP population, which is the basis for efficacy analysis, the upper limit of the 95% CI of the LDH AUC difference was about 12.3% of the previously identified placebo-controlled effect.

 The high frequency of hemoglobin level stabilization and dynamics of PNH RBC clone size act as additional confirmation of the efficacy of therapy due to a decrease in hemolytic activity. The number of episodes of breakthrough hemolysis and thrombotic complications, as well as the patient needs for pRBC transfusion, was insignificant and did not reveal significant differences between the Biosimilar and Originator. At the same time, the subjective state of the patients, investigated using the FACIT-Fatigue and EORTC QLQ-C30 questionnaires, confirmed the stability of laboratory and clinical data.

Extremely high variability of the baseline concentration of the medicinal product in both groups was noted during the analysis of the pharmacokinetic parameters: the coefficient of variation (CV) was 94.6% in Group A and 82.8% in Group B, respectively. The results obtained correspond to the conclusions of existing publications on the high variability of eculizumab, the concentration of which depends on the level of MAC and complement component C5, as well as on the age and sex of the patient population [19]. A decrease in medicinal product concentration variability was reported in both groups at subsequent time points, although the coefficient of variation at some points was significant and exceeded 50%, which can be explained by the achievement of stable complement inhibition. The mean concentration of eculizumab 5 min before the administration of the Biosimilar or Originator at all the visits before and in the steady state exceeded 35 μg/mL which is the minimum concentration sufficient enough for complete inhibition of intravascular hemolysis in patients with PNH [20, 21]. Also, high variability of individual pharmacokinetic parameters may be due to their significant dependence on the patient’s body weight [22].

According to pharmacodynamic studies, the concentration of MAC decreases after the end of the product infusion, then gradually increases and reaches its maximum at the end of the dosing interval, which confirms the pathophysiological activity of the medicinal product [23].

The overall safety profile of the Biosimilar was consistent with that of the Originator, and no cases of meningococcal or other severe infection or unexpected ADRs were reported. The proportion of patients with registered ADRs and the dynamics of laboratory and instrumental examination data in both treatment groups were comparable. New cases of ADA formation were not registered during the study, which indicates the absence of significant differences in safety parameters and is consistent with similar studies of biosimilars [24].

The main limitation of the clinical study was the low number of PNH patients, as well as the orphan status of eculizumab, which complicates the formation of a comparison group. These limitations influenced the choice of study design, efficacy endpoints, and sample size.

The use of a noninferiority design was justified by the fact that no substantial and clinically significant excess of efficacy was expected for the Biosimilar compared to the Originator when using the same regimen since the clinical efficacy of eculizumab is primarily determined by the minimum concentration sufficient to inhibit the activity of the terminal complement complex. According to the results of clinical studies of the Originator, this value in PNH patients is about 35 μg/mL [16, 24]. The noninferiority design is often used in comparative studies of biosimilars as well [25].

Thus, long-term use of eculizumab Biosimilar leads to successful inhibition of the complement hemolytic activity in PNH patients. The assessment of the efficacy and safety of the Biosimilar showed no less efficacy and comparability in terms of safety with the Originator, which is necessary to confirm its biosimilarity to the original medicinal product.

## Supplementary Information

Below is the link to the electronic supplementary material.

Supplementary file1 (22.6 KB)
