# Supplementary material for: Randomized multicenter noninferiority phase III clinical trial of the first biosimilar of eculizumab
Source: Ann Hematol. 2021 Aug 16;100(11):2689–98. doi: 10.1007/s00277-021-04624-7 (PMC8510904; doi:10.1007/s00277-021-04624-7)
Supplement: Supplementary file 1 — Supplementary file1 (22.6 KB) [file 277_2021_4624_MOESM1_ESM.docx]

1. **Supplementary methods**

***Investigations***

Blood samples for evaluation of the pharmacokinetic (PK) parameters were taken in the beginning and at the end of the study (at Visits 3–9 and 12–17) 5 minutes before the medicinal product administration and 1 hour ± 5 minutes after the end of infusion; at Visit 10: 5 minutes before the medicinal product administration, immediately after the end of infusion, 1 hour ± 5 minutes, 24 hours ± 30 minutes, 48 hours ± 2 hours, 96 hours ± 2 hours, 168 hours ± 2 hours, 240 hours ± 2 hours after the end of infusion; at Visit 11: immediately before the medicinal product administration (336 hours ± 2 hours after the end of infusion at Visit 10) and 1 hour ± 5 minutes after the end of infusion.

During the study, the patients underwent physical examination, anthropometry, evaluation of vital signs, ECG, Doppler echocardiography with an assessment of the average pressure in the pulmonary artery and the degree of pulmonary hypertension. The European Organization for Research and Treatment of Cancer Quality of Life Questionnaire (EORTC QLQ-C30) and a Functional Assessment of Chronic Illness Therapy-Fatigue Scale (FACIT-Fatigue) have been filled out by patients. Blood samples were taken to analyze the PNH clone, perform clinical and biochemical blood assays, coagulogram and D-dimer studies, urine samples were taken for urinalisys.

***Study Oversight***

The study was developed by the sponsor (JSC GENERIUM) and agreed with the researchers, authors of the publication. The data collection was performed by the sponsor's personnel using products from ENNOV CLINICAL S.A.S. Statistical and medical analysis of the data, as well as the preparation of the final report of the study, were performed by the sponsor. The sponsor guarantees the completeness, accuracy of the data and analysis performed and confirms that the publication complies with the protocol and other study documents. All authors had access to study results, manuscript drafts, and approved the final manuscript. The study protocol and the patient's informed consent form were approved by the Ethics Council of the Ministry of Health of the Russian Federation, as well as by independent ethical committees of research sites. The study was conducted in accordance with the ethical principles of the Declaration of Helsinki by the World Medical Association (WMA) (2013), the standards of the Guideline for Good Clinical Practice (GCP) of the International Conference on Harmonization of technical requirements for registration of pharmaceutical products for human use (ICH) to monitor patient interests and safety.

***End Points***

In this study, breakthrough hemolysis was defined as at least one new episode or worsening of at least one previously observed sign of intravascular hemolysis (fatigue; hemoglobinuria; abdominal pain; shortness of breath; severe vascular complications, including thrombosis; dysphagia; or erectile dysfunction) with elevated LDH after a previous decrease during treatment. During maintenance therapy with the Biosimilar, a comparative assessment of changes in hemoglobin, PNH clone size, and a number/proportion of patients with stable hemoglobin levels was performed. Additionally, pharmacokinetic and pharmacodynamic parameters were evaluated, as well as safety data.

***Statistical analysis***

The population of all patients included in the study (FAS-population, full analysis set) was used to describe primarily the initial characteristics, including demographic and anthropometric indicators of patients. Efficacy analysis was performed in all patients who completed the study without significant deviations from the protocol (per protocol (PP) population). During the study, the sufficient number of patients was randomized in order to detect at least half of the LDH AUC difference obtained previously between the investigational medicinal product and placebo (Δ = [363,740 – 62,471]/2 = 150,635 U/L*days), with a power of 80% and at a one-sided significance level of 0.025. The χ2 criterion or, if necessary, Fisher's exact test (if the expected frequency in any of the cells is less than 5) was used to analyze the secondary efficacy parameters, which are categorical variables.

1. **Supplementary results**

In Group A, 1 patient (6.3%) needed 2 transfusions, another 1 patient (6.3%) needed 3 or more transfusions during the study. In Group B, 2 patients (14.3%) required 2 transfusions, and 3 or more red blood cell transfusions were performed in 1 patient (7.1%).

1. **Supplementary tables**

***Table 1: Adverse Reactions (ADRs) by system organ class, preferred terms, and treatment groups***

| Appearance | Group A (Biosimilar) | | | Group B  (Originator) | | | p |
| --- | --- | --- | --- | --- | --- | --- | --- |
|  | N | % | Severity | N | % | Severity |  |
| Any PT | 3/16 | 18.8 | Grade | 2/16 | 12.5 | Grade | 1.000 |
| *INVESTIGATIONS* | | | | | | | |
| Positive Direct Coombs test | 1/16 | 6.3 | 3 | 0/16 | 0.0 | - | 1.000 |
| GFR decrease | 1/16 | 6.3 | 3 | 0/16 | 0.0 | - | 1.000 |
| Pulmonary artery pressure increase | 0/16 | 0.0 | - | 1/16 | 6.3 | 1 | 1.000 |
| *BLOOD AND LYMPHATIC SYSTEM DISORDERS* | | | | | | | |
| Neutropenia | 1/16 | 6.3 | 2 | 1/16 | 6.3 | 3 | 1.000 |
| Leukopenia | 0/16 | 0.0 | - | 1/16 | 6.3 | 3 | 1.000 |
| *INFECTIONS AND INFESTATIONS* | | | | | | | |
| Infection | 0/16 | 0.0 | - | 1/16 | 6.3 | 4 | 1.000 |
| Chronic pyelonephritis | 1/16 | 6.3 | 2 | 0/16 | 0.0 | - | 1.000 |
| Urinary tract infection | 1/16 | 6.3 | 2 | 0/16 | 0.0 | - | 1.000 |
| *RENAL AND URINARY DISORDERS* | | | | | | | |
| Chronic kidney disease | 1/16 | 6.3 | 1 | 0/16 | 0.0 | - | 1.000 |
| Proteinuria | 1/16 | 6.3 | 2 | 0/16 | 0.0 | - | 1.000 |
| *GENERAL DISORDERS AND ADMINISTRATION SITE CONDITIONS* | | | | | | | |
| Edema at the injection site | 1/16 | 6.3 | 1 | 0/16 | 0.0 | - | 1.000 |
| *METABOLISM AND NUTRITION DISORDERS* | | | | | | | |
| Hyperkalemia | 1/16 | 6.3 | 3 | 0/16 | 0.0 | - | 1.000 |
| Notes:  The results are presented in the following format: the number of subjects with reported AEs, the percentage of the Safety Population in this group.  The subject with the strongest relationship of AE with the investigational medicinal product observed in them was included in the analysis.  PT — preferred term | | | | | | | |
